# Optimizing Mechanical Ventilation Strategies in ARDS: The Role of Driving Pressure and Low Tidal Volume Ventilation

**DOI:** 10.1155/ccrp/8857930

**Published:** 2025-07-26

**Authors:** Vladislav Muldiiarov, Keely L. Buesing

**Affiliations:** Department of Surgery, Division of Acute Care Surgery, University of Nebraska Medical Center, Omaha, Nebraska, USA

**Keywords:** acute respiratory distress syndrome (ARDS), driving pressure, mechanical ventilation, positive end-expiratory pressure (PEEP), tidal volume, transpulmonary pressure, ventilator-induced lung injury (VILI)

## Abstract

**Importance:** Mechanical ventilation is indispensable for the management of acute respiratory distress syndrome (ARDS), yet suboptimal ventilator settings can exacerbate lung injury. There is growing evidence that lung-protective ventilation strategies reduce ventilator-induced lung injury (VILI) and improve outcomes. Understanding the role of key parameters, such as driving pressure and tidal volume, is essential for optimizing patient care.

**Observations:** This narrative review synthesizes the evidence underpinning the evolution of lung-protective ventilation strategies in ARDS, focusing on the importance of low tidal volume ventilation and the monitoring of driving pressure. A targeted literature search was performed in PubMed, Embase, The Cochrane Library, Google Scholar, and Web of Science up to April 2025, focusing on adult ARDS. Original research studies (randomized controlled trials, retrospective and prospective cohort studies) and meta-analyses published in English were included.

**Conclusions and Relevance:** Evidence supports adopting lung-protective strategies, including low tidal volume ventilation and careful driving pressure monitoring, to reduce VILI and improve survival in ARDS patients. By integrating these evidence-based principles into mechanical ventilation management, clinicians can enhance patient outcomes, reduce iatrogenic harm, and advance the overall quality of ARDS care.

## 1. Introduction

Acute respiratory distress syndrome (ARDS) is a severe, diffuse inflammatory lung injury presenting a life-threatening condition in critically ill patients. It is characterized by rapid onset of respiratory failure, hypoxemia refractory to oxygen therapy, and bilateral pulmonary infiltrates on imaging [1, 2]. At the microscopic level, ARDS involves inflammation-mediated disruption of the alveolar-capillary barrier, leading to increased permeability, pulmonary edema formation, decreased alveolar fluid clearance, alveolar collapse, reduced lung compliance, and increased pulmonary vascular resistance. These changes result in gas exchange abnormalities due to shunting and ventilation–perfusion mismatch [3, 4]. Despite advancements in understanding the pathophysiology of acute lung injury and improvements in medical technology, the mortality rate associated with ARDS remains high, reaching up to 40% [5, 6]. ARDS is a significant health concern, affecting approximately 10% of patients admitted to intensive care units (ICUs) and up to 23% of those requiring mechanical ventilation [7, 8].

## 2. A Contemporary Perspective on ARDS

The clinical definition of ARDS has evolved since its initial description in 1967 by Ashbaugh et al. [9]. The American-European Consensus Conference (AECC) established criteria in 1994, which were later refined in the Berlin definition of 2012 [10–12]. The current criteria can be summarized as follows: acute onset of respiratory failure within 1 week of a known clinical insult, bilateral opacities on chest imaging or ultrasound not fully explained by effusions, lobar/lung collapse or nodules, and respiratory failure not fully explained by cardiac failure or fluid overload. Oxygenation impairment is classified as follows: for nonintubated ARDS, it is defined by a PaO_2_/FiO_2_ ratio < 300 mmHg or an SpO_2_/FiO_2_ ratio < 315 (if SpO_2_ < 97%) on high-flow nasal oxygen with a flow rate > 30 L/min or noninvasive ventilation/CPAP with at least 5 cm H_2_O of PEEP. For intubated ARDS, “mild” is characterized by a PaO_2_/FiO_2_ ratio of 200–300 mmHg or an SpO_2_/FiO_2_ ratio ≤ 315 (if SpO_2_ < 97%). “Moderate” is defined by a PaO_2_/FiO_2_ ratio of 100–200 mmHg or an SpO_2_/FiO_2_ ratio ≤ 235, and “severe” by a PaO_2_/FiO_2_ ratio < 100 mmHg or an SpO_2_/FiO_2_ ratio ≤ 148 [13].

Mechanical ventilation is a cornerstone in managing ARDS, providing life-sustaining support [14]. However, it can also induce ventilator-induced lung injury (VILI) due to excessive stress and strain from alveolar overdistension and repetitive opening and closing of unstable lung units [15–17]. This mechanical trauma leads to a cascade of inflammatory responses, including fibrogenesis, elastogenesis, and activation of matrix metalloproteinases, which can exacerbate lung injury, delay recovery, and worsen clinical outcomes [18–20].

Numerous studies have emphasized the pivotal role of lung-protective ventilation strategies in the management of ARDS. These strategies focus on optimizing key respiratory mechanics, including tidal volume, driving pressure, lung compliance, flow rate, positive end-expiratory pressure (PEEP), and respiratory rate. Specifically, low tidal volume (LTV) ventilation has been shown to reduce mortality by minimizing VILI [21–23]. Furthermore, recent evidence indicates that driving pressure (Δ*P*), the difference between plateau pressure and PEEP, may be a more accurate predictor of mortality than tidal volume alone [24]. Understanding and optimizing Δ*P* could therefore enhance patient outcomes.

This review aims to explore current evidence-based findings regarding Δ*P* and LTV ventilation in the management of ARDS. By critically analyzing recent studies and clinical trials, we seek to provide insights into optimal ventilation strategies that can minimize VILI and improve survival rates in patients with ARDS.

## 3. Methods

This narrative review is based on a targeted literature search conducted in PubMed, Embase, the Cochrane Library, Google Scholar, and Web of Science between December 2024 and April 2025 (Table 1). The search was performed in English using a combination of controlled vocabulary and free-text keywords, including “acute respiratory distress syndrome (ARDS),” “mechanical ventilation,” “driving pressure,” “lung-protective ventilation,” “low tidal volume,” and “ventilator-induced lung injury (VILI).” The review included original peer-reviewed studies, randomized controlled trials, prospective and retrospective cohort studies, as well as meta-analyses focusing on adult patients with ARDS. Studies were excluded if they were not published in English, lacked peer review, provided insufficient methodological detail, or focused solely on pediatric populations or highly specialized surgical contexts. The selection process involved an initial screening of titles and abstracts, followed by full-text review to confirm eligibility. Emphasis was placed on the most recent and clinically relevant studies that examined the effects of LTV ventilation and driving pressure strategies in ARDS management. As this is a narrative, nonsystematic review, no formal risk of bias assessment (e.g., using RoB2 or ROBINS-I) was performed.

## 4. The Historical Evolution of Lung-Protective Mechanical Ventilation

Implementing lung-protective ventilation is essential in the treatment of patients with ARDS and plays a pivotal role in enhancing clinical outcomes. The main objective of this approach is to prevent the overinflation of aerated lung regions. Excessive stretching of these areas can cause damage to both the pulmonary endothelium and epithelium, leading to a cascade of adverse effects such as pulmonary inflammation, atelectasis, and hypoxemia.

The exploration of protective ventilation began in 1967, initially focusing on optimizing gas exchange [9, 25]. In 1990, Hickling demonstrated that a mechanical ventilation strategy that reduced peak inspiratory pressure (PIP) and tolerated hypercapnia could decrease mortality in ARDS patients [26].

A pivotal study by the ARDS Network in 2000 further emphasized the importance of lung-protective ventilation strategies [23]. This landmark trial showed that using lower tidal volumes (6 mL/kg of predicted body weight [PBW]) significantly reduced hospital mortality and shortened the duration of mechanical ventilation. Prior to this, conventional ventilation methods aimed to enhance arterial oxygenation through higher tidal volumes (upwards of 10–12 mL/kg PBW), often resulting in alveolar overdistension. This overdistension contributed to stretch-induced damage of the alveolar endothelium and amplified the innate inflammatory response, exacerbating the underlying mechanisms of ARDS. The protocol derived from the ARDSNet trial provided compelling evidence that minimizing alveolar stretch injury with LTV improves survival, leading to its adoption as the mainstay of ventilatory management in ARDS.

Despite these advancements, the LUNG SAFE study, a large multinational prospective cohort study on severe respiratory failure published in 2016, revealed both an underdiagnosis of ARDS and widespread noncompliance with lung-protective ventilation strategies [7]. The study found that fewer than two-thirds of patients with ARDS received tidal volumes less than 8 mL/kg PBW.

Currently, lung-protective mechanical ventilation strategies often include the following components: LTV ventilation, reduced PIP, plateau pressure, Δ*P*, the application of higher PEEP, permissive hypercapnia, and conservative oxygen targets [27–31].

## 5. ARDS-Net Ventilation Strategy

The first major randomized clinical trial to provide direct evidence of the benefits of low-tidal-volume ventilation in ARDS patients was published by Amato et al. [21]. This trial compared conventional ventilation to a low-tidal-volume, “protective ventilation” strategy in 53 patients with early ARDS. The study demonstrated that the protective ventilation approach significantly reduced 28-day mortality and led to better outcomes, such as higher rates of successful weaning from mechanical ventilation and a lower incidence of barotrauma, marking a pivotal shift in ARDS treatment strategies. However, several small, randomized trials conducted during and since that period have not demonstrated a benefit of LTV ventilation in patients with acute lung injury [32–34].

The original ARDS Network (ARDSNet) LTV approach (i.e., 6 mL/kg PBW) aims to protect the normal lung from overdistension injury (volutrauma) [23]. Persistently collapsed lung tissue is allowed to “rest” by remaining unventilated. This LTV approach also strives to keep plateau pressure (*P*plat) less than 30 cm H_2_O with the application of PEEP guided by oxygenation necessary to prevent progressive loss of end-expiratory lung volume. The ARDSNet trial demonstrated that ventilation with lower tidal volumes (6 mL/kg PBW) significantly reduced mortality and increased ventilator-free days in patients with acute lung injury and ARDS. Compared to traditional tidal volumes (12 mL/kg PBW), the lower tidal volume strategy decreased mortality from 39.8% to 31% (*p*=0.007) and increased the number of ventilator-free days within the first 28 days (*p*=0.007). These results underscore the importance of lung-protective ventilation strategies in improving outcomes for patients with ARDS [23].

Despite the proven benefits of the ARDSNet trial in promoting lung-protective ventilation, adoption remains low, with less than 50% of patients with acute lung injury receiving evidence-based ventilation. Factors such as metabolic acidosis, lack of formal protocols, and difficulties calculating predicted body weight have been associated with the underuse of LTV, highlighting barriers to the implementation of evidence-based practice [35–41].

In an effort to evaluate the effectiveness of LTV strategies further, several studies have been conducted with varying results. Walkey et al. performed a systematic review and meta-analysis comparing LTV strategies to conventional methods in patients with ARDS. Analyzing seven randomized trials involving 1,481 patients, they found no statistically significant reduction in mortality with LTV alone (33.6% vs. 40.4%; RR, 0.87; 95% CI, 0.70–1.08). However, when LTV was combined with high PEEP, mortality was significantly reduced (RR, 0.80; 95% CI, 0.66–0.98), suggesting a synergistic benefit [42]. Similarly, Simonis et al., in the PReVENT trial (*n* = 961), compared an LTV strategy to an intermediate tidal volume strategy in patients without ARDS and found no significant difference in patient outcomes between the two ventilation strategies [43]. The contradictory results may be attributed to two key factors. First, the high heterogeneity of the patient population could have diminished the detectable effect of ventilation strategies. Second, the differences in tidal volume between groups were minimal, especially after the first day, as most patients (65% in the LTV group and 64% in the intermediate group) received tidal volumes within the 6–10 mL/kg PBW range [43].

Hospital adherence to LTV in patients with ARDS has also been explored. LeSieur et al. evaluated adherence across 110 hospitals and found that 73% of patients received LTV (defined as 4–8 mL/kg PBW) over the first 72 h of mechanical ventilation. Despite variations in hospital adherence rates (ranging from 13% to 95%), the study did not find an association between hospital adherence to LTV and risk-adjusted mortality rates [44].

Focusing on the predictors of mortality, Dianti et al. conducted a meta-regression analysis of nine trials involving 4,731 patients with ARDS. The study demonstrated that LTV and Δ*P* in lung-protective ventilation strategies significantly reduced mortality. Variations in tidal volume, Δ*P*, and mechanical power were all associated with increased mortality; however, tidal volume and Δ*P* were better predictors of mortality than mechanical power [45]. This highlights the potential importance of driving pressure as a target for ventilation strategies.

In the emergency department setting, De Monnin et al. performed a systematic review and meta-analysis that included 12,912 patients from 11 studies. The use of LTV ventilation in emergency department patients was associated with significant improvements in clinical outcomes, such as reduced mortality (26.5% vs. 31.1%, OR 0.80) and a lower incidence of ARDS (4.5% vs. 8.3%, OR 0.57). Additionally, LTV ventilation was linked to shorter ICU and hospital stays, more ventilator-free days, and reductions in tidal volume in both the ED and ICU settings. Despite the low quality of evidence, these findings highlight the potential value of implementing LTV ventilation early in patient care [46].

Recently, a randomized controlled trial by Tongyoo et al. further examined this comparison by assessing the effectiveness of LTV ventilation versus a driving pressure–limited strategy in preventing VILI in 126 adults with acute respiratory failure. The study found no significant difference between the two strategies in reducing lung injury 7 days after the initiation of mechanical ventilation [47]. One possible reason for the lack of a statistically significant difference is that the duration of Δ*P* exceeding 15 cm H_2_O was similar in both groups, whereas it should have been lower in the limited Δ*P* ventilation group. This could be attributed to delayed physician interventions, which may have occurred only after airway pressures had already risen, rather than proactively adjusting ventilation settings to maintain lower Δ*P*.

### 5.1. Hypercapnia: A Double-Edged Sword in Lung Protection

In clinical practice, protective ventilation can elevate arterial CO_2_ levels, resulting in hypercapnia. This phenomenon, which arises from LTV to achieve PV, is often accepted to minimize VILI [48]. Nevertheless, the overall impact of hypercapnia remains controversial. Animal-based studies suggest that hypercapnic acidosis can mitigate lung damage by downregulating nuclear factor kappa B, reducing nitric oxide synthesis, and offering anti-inflammatory and surfactant-related benefits [49, 50]. In contrast, an excessive rise in CO_2_ has also been linked to adverse effects, including diminished alveolar epithelial restoration, weakened neutrophil and innate immune function, pulmonary hypertension, and right ventricular strain [51–53].

In patients with hypercapnic respiratory failure, LTV can exacerbate dead space ventilation and lead to alveolar hypoventilation [23]. To compensate, it is often necessary to increase respiratory rate in order to maintain adequate CO_2_ clearance, a step that may introduce additional challenges related to patient comfort and sedation.

Clinical findings reflect the complexity of hypercapnia. For example, in a study of 1,899 ARDS patients, Nin et al. observed that severe hypercapnia (PCO_2_ > 50 mmHg) within the first 48 h of ICU admission was independently associated with elevated ICU mortality and complications such as barotrauma, renal dysfunction, and cardiovascular instability [54]. Mortality rose further among individuals exhibiting compensated hypercapnia or hypercapnic acidosis compared to those with normocapnia and normal pH. Likewise, a large multicenter investigation by Tiruvoipati et al. (*N* = 252,812) revealed a significantly higher adjusted hospital mortality in patients with compensated hypercapnia or hypercapnic acidosis, with mortality odds progressively increasing in parallel with PCO_2_ levels [55].

These conflicting data suggest that hypercapnia may exert protective effects when it naturally accompanies LTV but can become detrimental when it is severe or superimposed on an already reduced tidal ventilation (approximately 6 mL/kg predicted body weight). Consequently, permissive hypercapnia remains a key component of lung-protective strategies but demands meticulous patient selection, vigilant CO_2_ monitoring, and carefully individualized ventilatory management.

## 6. Driving Pressure

Δ*P* is calculated as the difference between plateau pressure (*P*plat) and PEEP. Plateau pressure is recorded at the end of an inspiratory pause during volume-controlled constant flow ventilation or at the end of inspiration during pressure-controlled ventilation. In the absence of patient respiratory effort, driving pressure represents the pressure above PEEP exerted on the entire respiratory system to achieve tidal ventilation [56].

### 6.1. Evidence Supporting Driving Pressure as a Predictor of Outcomes

One of the earliest explorations of Δ*P* as a key factor in lung protection comes from the study by Amato et al., which assessed a protective ventilation strategy for patients with ARDS [21]. This study emphasized the importance of reducing alveolar collapse and preventing overdistention by employing lower tidal volumes and maintaining Δ*P* below 20 cm H_2_O. The findings showed a significant improvement in 28-day survival rates compared to conventional ventilation strategies, with added benefits such as reduced barotrauma and higher rates of successful weaning from mechanical ventilation. However, despite these short-term advantages, the protective strategy did not yield a statistically significant improvement in overall survival to hospital discharge. Nevertheless, this study laid the foundation for the concept of Δ*P* as a critical parameter in minimizing VILI and enhancing early recovery in ARDS patients.

Building upon this foundation, Amato and colleagues in 2015 conducted a comprehensive analysis of nine randomized controlled trials involving 3,562 ARDS patients [24]. Through multilevel mediation analysis, they identified Δ*P* as the variable most strongly associated with survival, even within the context of LTV ventilation strategies. Their findings revealed a significant correlation between higher Δ*P* and increased mortality risk (RR of death: 1.36, 95% CI: 1.17–1.58, *p* < 0.001), with Δ*P* exceeding 15 cm H_2_O particularly linked to elevated mortality rates.

A comparison between LTV ventilation and Δ*P* in ARDS patients was conducted by Guérin et al., who analyzed data from 787 patients included in two independent randomized controlled trials [57]. These trials evaluated various adjunctive ventilation strategies, including those used in the LTV_t_ group from the ARDSNet study. Their analysis demonstrated that Δ*P* was significantly associated with patient outcomes after adjusting for confounding factors. Survival was notably higher in patients with Δ*P* ≤ 13 cm H_2_O on the first day, as well as in those with mechanical power ≤ 12 J/min and *P*plat < 23 cm H_2_O. Furthermore, Δ*P* exhibited a stronger correlation with survival compared to PEEP and tidal volume, whereas neither PEEP nor tidal volume was associated with mortality in any of the models analyzed.

Further supporting these conclusions, a systematic review and meta-analysis by Aoyama et al., encompassing seven studies and over 6,062 patients receiving mechanical ventilation for ARDS, demonstrated a clear association between higher Δ*P* and increased mortality [58].

Similarly, Sahetya et al. explored the effect of Δ*P* on patient outcomes in acute ARDS within a large multicenter cohort of 1,132 patients [59]. They found that higher Δ*P* was independently associated with increased hospital mortality in patients with ARDS, reinforcing the critical role Δ*P* plays in VILI. This study highlights the importance of optimizing Δ*P* through lung-protective strategies, such as reducing tidal volumes and adjusting PEEP. The results are consistent with previous epidemiological studies, which have shown improved outcomes with LTV ventilation compared to higher tidal volumes, as well as lower Δ*P* and *P*plat in patients [8, 60].

Expanding the scope beyond ARDS patients, Lanspa et al. examined the effect of Δ*P* on mortality in both ARDS and non-ARDS patients receiving mechanical ventilation (*n* = 2641) [61]. The study found that in patients with ARDS, Δ*P* was associated with increased 30-day mortality (OR 1.1, *p* < 0.001), suggesting that the detrimental effects of elevated Δ*P* are particularly pronounced in this population.

Additionally, Blondonnet et al. conducted a multicenter observational study involving 221 intubated ICU patients with at least one ARDS risk factor to investigate the link between elevated Δ*P* and the onset of ARDS [62]. They found that patients who developed ARDS within 7 days had significantly higher baseline Δ*P* than those who did not (12.5 ± 3.1 vs. 9.8 ± 3.4 cm H_2_O, *p*=0.0001). This association remained significant even after adjusting for other factors like tidal volume and illness severity, indicating that elevated Δ*P* may contribute to the development of ARDS in at-risk patients.

In a multicenter observational study, Roca et al. evaluated the impact of Δ*P* on patients (*n* = 1575) without ARDS undergoing mechanical ventilation. The study demonstrated that a higher Δ*P* on the first day of ventilation was significantly associated with the subsequent development of ARDS. Specifically, for each 1 cm H_2_O increase in Δ*P*, the risk of developing ARDS increased by 10% (OR 1.12, 95% CI 1.07–1.18, *p* < 0.001). Subjects with a Δ*P* greater than 12 cm H_2_O were found to have a notably higher risk of ARDS [63]. The prospective controlled trial (*n* = 110) by Hamama et al. demonstrated a significant decrease in mortality and ventilator-free days with Δ*P*-directed ventilation, employing a limit of 14 cm H_2_O through PEEP adjustments for patients ventilated with 4–6 mL/kg [64].

In a study by Goodwin et al., the effects of Δ*P* and respiratory system elastance on outcomes in patients (*n* = 4223) with ARDS were explored using a real-world electronic health record dataset. The study found that elevated Δ*P* above 15 cm H_2_O was significantly associated with increased mortality and fewer ventilator-free days, independent of lung-protective ventilation adherence and severity of illness [65].

A multicenter randomized trial by Park et al. involving 1,170 patients undergoing lung resection surgery assessed whether targeting lower airway Δ*P* could reduce postoperative pulmonary complications [66]. Patients were randomized to either a driving pressure–guided ventilation strategy with alveolar recruitment and individualized PEEP to minimize Δ*P*, or to conventional protective ventilation with fixed PEEP of 5 cm H_2_O. The driving pressure group achieved significantly lower mean Δ*P* (7.1 vs. 9.2 cm H_2_O) and showed improved intraoperative lung compliance and oxygenation. However, there was no significant difference in the incidence of pulmonary complications within 7 days postoperatively between the two groups (40.5% vs. 42.8%, *p*=0.42). This suggests that although Δ*P*-guided ventilation enhances intraoperative pulmonary mechanics, it does not reduce postoperative pulmonary complications compared to conventional LTV ventilation in this surgical population. Additionally, several studies have shown that high intraoperative Δ*P* and changes in PEEP levels that lead to increased Δ*P* are associated with a higher incidence of postoperative pulmonary complications [67–72].

A recent systematic review and meta-analysis by Muñoz et al. evaluated the efficacy of personalized ventilation strategies guided by Δ*P* adjustments in patients with ARDS [73]. Analyzing 13 randomized controlled trials involving 1,255 patients, the study found no significant mortality benefit for Δ*P*-guided ventilation compared to conventional lung-protective strategies (RR 0.61, 95% CI 0.29–1.30), and no relevant improvements in ventilator-free days or gas exchange parameters. These findings highlight the uncertainty surrounding Δ*P*-targeted approaches in controlled trial settings.

In contrast, large-scale observational data suggest potential clinical value in Δ*P* optimization. Urner et al. analyzed a cohort of 12,865 mechanically ventilated patients and found that those managed with a Δ*P* < 15 cm H_2_O had lower mortality (18.1%) compared to standard care (20.1%), indicating a possible benefit of Δ*P*-limited strategies in real-world practice [74]. Supporting this, Costa et al. studied 4549 patients with ARDS and demonstrated that while mechanical power was associated with mortality, Δ*P* and RR were the most powerful predictors [75]. Notably, the effect of Δ*P* on mortality was fourfold greater than that of RR, and a simplified model incorporating only these two parameters performed as well as models based on full mechanical power calculations. Expanding the scope beyond ARDS, Xie et al. evaluated the impact of initial DP in a broader population of 907 ICU patients with acute hypoxemic respiratory failure (AHRF) using the MIMIC-IV database. Their results showed that an initial DP > 12 cm H_2_O was independently associated with higher mortality at 28, 90, and 180 days. This association was particularly strong in patients with a P/F ratio ≤ 150 mmHg [76].

Age-related structural and functional changes in the lung—such as enlarged alveolar spaces, diminished elasticity, declining immune function, and the accumulation of senescent cells—can further complicate the course of ARDS in older adults [77, 78]. Although limited studies have specifically assessed the impact of aging on ∆*P*, it is plausible that reduced lung compliance in elderly patients leads to higher ∆*P* values, increasing susceptibility to VILI. Advanced age is consistently associated with worse outcomes in the ICU, with multiple studies identifying it as a key predictor of mortality [79, 80]. A large-scale analysis by Papoutsi et al. of 4567 ARDSNet patients further underscored this association, revealing an age-dependent relationship between ∆*P* and mortality, particularly in individuals over 80 years old. In this subgroup, a ∆P threshold of 11 cm H_2_O was significantly correlated with increased mortality (*p* < 0.01) [81].

Obesity presents additional challenges in the mechanical ventilation of ARDS patients, as altered respiratory mechanics may modify the relationship between ∆*P* and outcomes. Patients with obesity often exhibit increased chest wall elastance, lower transpulmonary pressures, and reduced overall compliance, which can make standard ∆*P* calculations less reliable. A retrospective study by De Jong et al. involving 362 ARDS patients—100 with obesity and 262 without—found no significant association between ∆*P* during the first 24 h of ventilation and 90-day mortality in obese individuals, suggesting that ∆*P* may not be a universally applicable parameter across all patient populations [82].

### 6.2. Challenges in Achieving Optimal Driving Pressure in the Presence of Patient–Ventilator Asynchrony

Achieving optimal driving pressure–limited ventilation is inherently complex, particularly in patients who exhibit spontaneous breathing efforts. Patient–ventilator asynchrony (PVA) represents a major barrier to maintaining lung-protective ventilation, and its presence can significantly impact both measured and actual Δ*P*, confounding attempts to optimize ventilator settings. Among the various forms of PVA, double triggering, reverse triggering, and ineffective effort (wasted efforts) are particularly relevant in the context of driving pressure–limited ventilation. Each of these phenomena introduces unique challenges that may paradoxically increase lung stress and contribute to VILI [83–85].

#### 6.2.1. The Impact of Double Triggering on Driving Pressure and Tidal Volume

Double triggering—one of the most common forms of PVA—occurs when the patient's inspiratory effort persists beyond the ventilator's set inspiratory time, triggering a second mechanical breath before the patient has fully exhaled from the first. This breath stacking effectively doubles the delivered tidal volume, substantially increasing transpulmonary pressure (P_l_) and the risk of volutrauma [82]. Notably, double triggering is more prevalent in ARDS patients undergoing LTV ventilation, where intense respiratory drive due to unmet ventilatory demand results in prolonged inspiratory efforts that exceed ventilator cycle times [83].

From a Δ*P* standpoint, double triggering artificially lowers the calculated Δ*P* if it is assessed during a passive inspiratory hold, as the measured plateau pressure may not fully capture the dynamic summation of tidal volumes. However, the true driving pressure (Δ*P* = *P*plat − PEEP) may be significantly higher in real-time breathing cycles, increasing lung stress and the potential for lung injury. This discrepancy underscores the importance of continuous rather than snapshot monitoring of Δ*P* to detect occult breath-stacking and its clinical impact.

#### 6.2.2. Reverse Triggering: A Hidden Driver of Increased Driving Pressure

In deeply sedated or paralyzed patients, reverse triggering (entrainment) is another insidious mechanism by which ventilator-driven breaths inadvertently stimulate diaphragmatic contraction via neuromechanical coupling [85]. Although the precise pathophysiology is incompletely understood, reverse triggering can be identified by a subtle delay between ventilator-delivered inspiration and the onset of diaphragmatic contraction, which manifests as a negative deflection in esophageal and airway pressures.

This entrainment effect leads to unexpected increases in transpulmonary pressure and tidal volume, particularly in noncompliant lungs, as the patient's inspiratory effort adds to the ventilator-delivered breath. Reverse triggering has been associated with worsened lung injury through excessive mechanical power, a phenomenon that includes elevated pressure, increased cyclic stretch, and potential diaphragmatic injury [83]. Unlike traditional breath-stacking seen in double triggering, the entrained breath may be out-of-phase with the ventilator cycle, producing regional lung stress that is difficult to detect using conventional ventilator waveform analysis.

#### 6.2.3. Ineffective Efforts and the Risk of Patient Self-Inflicted Lung Injury (P-SILI)

Ineffective efforts, also known as wasted inspiratory efforts, occur when the patient attempts to trigger a breath but fails to meet the ventilator's triggering threshold, leading to an unrecognized inspiratory effort. This asynchrony is particularly concerning in the setting of elevated respiratory drive, where excessive inspiratory effort against a closed ventilator circuit can generate profound negative pleural pressure swings [85].

This phenomenon, known as P-SILI, may cause.

• Increased pulmonary vascular permeability, exacerbating pulmonary edema due to high capillary transmural pressures.
• Excessive lung stress, where negative pleural pressure increases the effective transpulmonary pressure, even in the presence of seemingly lung-protective tidal volumes.
• Nonuniform stress distribution, increasing local lung strain and worsening regional VILI [83–85].

Importantly, these increased negative swings in pleural pressure are not accounted for in traditional Δ*P* calculations, leading to an underestimation of lung stress. Even when ventilator settings appear optimized, these hidden pleural pressure changes may paradoxically elevate the true Δ*P* experienced by the lung beyond safe thresholds.

#### 6.2.4. Clinical Strategies to Mitigate PVA in Driving Pressure–Limited Ventilation

Given the complex interactions between PVA and Δ*P*, a multimodal approach is required to mitigate asynchrony while maintaining lung-protective strategies.

1. Advanced monitoring to accurately assess true driving pressure
  a. Esophageal pressure monitoring can help quantify actual transpulmonary pressure, distinguishing between ventilator-applied and patient-generated Δ*P* [85].
  b. Continuous monitoring of ventilator waveforms and automated detection algorithms for asynchrony may improve recognition and real-time intervention.
2. Targeted adjustments in ventilator settings
  a. For double triggering: increasing inspiratory time, adjusting inspiratory flow, and reducing tidal volume overshooting in volume-cycled modes may help limit unintended breath stacking.
  b. For reverse triggering: lowering mandatory respiratory rate, reducing sedation depth, and utilizing assist-control rather than fully controlled modes may minimize entrainment phenomena [83].
  c. For ineffective efforts/P-SILI: optimizing PEEP to minimize intrinsic PEEP, avoiding excessive respiratory drive (e.g., hypercapnia control), and considering proportional assist ventilation or neurally adjusted ventilatory assist modes can help mitigate excessive inspiratory effort [85].
3. Sedation and neuromuscular blockade in select patients
  a. Early neuromuscular blockade in patients with severe ARDS and high respiratory drive has been associated with reduced PVA, improved lung compliance, and lower mortality [83].
  b. Minimizing over-sedation may reduce the risk of reverse triggering, while sedation titration to patient comfort levels can help prevent double triggering.

#### 6.2.5. Conclusion: Balancing the Benefits and Risks of Driving Pressure–Limited Ventilation

While limiting Δ*P* remains an important goal in lung-protective ventilation, unrecognized PVA may paradoxically increase lung stress and injury. Double triggering, reverse triggering, and ineffective efforts all have the potential to distort measured Δ*P*, underestimating the actual mechanical forces applied to the lung. Careful integration of advanced monitoring, ventilator optimization, and patient-centered approaches is essential to safely apply driving pressure–guided ventilation without inadvertently exacerbating lung injury.

### 6.3. Barriers to Implementation of Driving Pressure and LTV Ventilation Strategies

Despite strong evidence supporting the use of Δ*P* and LTV strategies in ARDS, their consistent application in clinical practice remains challenging. Several key barriers must be addressed to optimize their adoption across different healthcare settings.

#### 6.3.1. Technological Limitations

One of the primary challenges in implementing driving pressure–guided ventilation is the need for advanced ventilators capable of accurately measuring and displaying plateau pressure and PEEP in real time. While modern ICU ventilators provide these parameters, older models or those in resource-limited settings may lack this functionality. Furthermore, real-time monitoring of transpulmonary pressure, which could enhance Δ*P* assessment, is not widely available and may require invasive esophageal manometry, limiting its feasibility in some settings.

#### 6.3.2. Clinician Resistance and Practice Variability

Despite the proven benefits of lung-protective ventilation, clinician adherence remains inconsistent. Studies have shown that compliance with LTV ventilation remains suboptimal, with less than 50% of eligible ARDS patients receiving appropriately adjusted tidal volumes. Factors contributing to this variability include:

• Concerns about patient comfort: reducing tidal volume often results in permissive hypercapnia, which may lead to increased dyspnea or agitation, requiring deeper sedation or paralysis.
• Perceived risks of hypoventilation: some clinicians hesitate to apply strict LTV targets due to concerns about hypercapnic acidosis, which can have hemodynamic consequences in critically ill patients.
• Lack of formal protocols: institutions without standardized ventilation protocols may rely on individual physician preference rather than guideline-based strategies.

Addressing these barriers requires ongoing clinician education, the development of automated decision-support tools, and clear institutional protocols to reinforce adherence to lung-protective strategies.

#### 6.3.3. Challenges in Specific Patient Populations

Implementation challenges are further magnified in certain patient subgroups:

• Elderly patients: aging lungs exhibit reduced elasticity, leading to increased alveolar collapse at lower airway pressures. Consequently, elderly patients may have higher intrinsic Δ*P* even at lower tidal volumes, increasing their susceptibility to VILI. Studies suggest that an individualized approach, with close monitoring of lung compliance and careful PEEP titration, may be necessary to balance protective ventilation with adequate gas exchange.
• Obese patients: obesity alters respiratory mechanics due to increased chest wall elastance and reduced functional residual capacity. Standard Δ*P* targets may not be directly applicable, as higher PEEP levels may be needed to maintain alveolar recruitment. In such cases, a personalized approach incorporating transpulmonary pressure monitoring may be more effective than a fixed tidal volume strategy.
• Patients with preexisting pulmonary disease: individuals with chronic obstructive pulmonary disease or interstitial lung disease may require deviations from strict LTV protocols due to preexisting abnormalities in lung compliance. These patients often develop intrinsic PEEP, which complicates the accurate assessment of Δ*P*. Ventilation strategies in these populations should prioritize minimizing dynamic hyperinflation while still aiming to reduce excessive tidal volumes.

#### 6.3.4. Strategies for Overcoming Implementation Barriers

To facilitate the broader adoption of evidence-based ventilation strategies, several steps can be taken:

• Integration of automated ventilator settings: emerging technologies allow for automated adjustments to maintain lung-protective targets, reducing the burden on clinicians.
• Multidisciplinary education and training: regular ICU training sessions on mechanical ventilation, with an emphasis on interpreting Δ*P*, could improve adherence.
• Institutional protocols and checklists: standardizing ventilator management strategies can reduce practice variability and ensure consistent application of lung-protective principles.

By addressing these barriers, clinicians can optimize the practical application of Δ*P* and LTV ventilation strategies, ensuring that evidence-based approaches translate into improved patient outcomes.

### 6.4. Limitations and Future Directions

While this narrative review synthesizes current evidence on Δ*P* and LTV ventilation in ARDS, it is essential to acknowledge several inherent limitations. First, the nonsystematic design of the analysis may lead to selection bias due to the absence of predefined eligibility criteria, structured screening procedures, and quantitative synthesis. As a result, certain relevant studies may have been overlooked, and the conclusions drawn remain contingent on the literature included. Moreover, despite our efforts to incorporate the most recent and influential investigations, the rapidly evolving landscape of ARDS research suggests that ongoing clinical trials and emerging data could refine—or potentially challenge—our interpretations. In addition, heterogeneity in study methodologies, patient populations, and ventilation strategies limits the generalizability of our findings, reinforcing the need for cautious application of these insights to clinical practice.

Recognizing these constraints underlines the importance of further high-quality, large-scale randomized controlled trials to compare ventilation strategies targeting Δ*P* against traditional LTV approaches. Advances in technologies such as real-time lung compliance monitoring or advanced imaging techniques may offer additional avenues to personalize ventilation for individual patients. Indeed, several clinical trials are underway or planned for 2024-2025 to evaluate various ventilatory techniques in adult ARDS patients [86, 87].

Concurrently, attention must turn to the persistent barriers that hinder broader adoption of evidence-based ventilation practices—foremost among them, the need for enhanced clinician education, standardized protocols, and decision-support tools that incorporate Δ*P* metrics at the bedside. Bridging the gulf between emerging research and frontline care is pivotal for improving clinical outcomes. Ultimately, through a confluence of scientific innovation and practical application, we can optimize mechanical ventilation for ARDS patients, thereby striking a more delicate balance between life-sustaining support and minimal iatrogenic harm.

## Figures and Tables

**Table 1:** Search strategy summary.

Items	Specification
Date of search	December to April 2025

Databases and other sources searched	PubMed, Embase, the Cochrane Library, Google Scholar, and Web of Science

Search terms used	Acute respiratory distress syndrome, mechanical ventilation, driving pressure, lung-protective ventilation, low tidal volume, and ventilator-induced lung injury.

Time frame	Available publications up to 4/30/2025

Inclusion and exclusion criteria	Inclusion: English language publications, retrospective studies, prospective studies, randomized controlled trials, systematic review, and meta-analysis
	Exclusion: non-English publications, nonscientific documents, review, narrative review

Selection process	Following review and discussion among authors

## Data Availability

Data sharing is not applicable to this article as no datasets were generated or analyzed during the current study.

## References

[1] Cave C., Samano D., Sharma A. M., Dickinson J., Salomon J., Mahapatra S. (2024). Acute Respiratory Distress Syndrome: A Review of ARDS Across the Life Course. *Journal of Investigative Medicine*.

[2] Matthay M. A., Zemans R. L., Zimmerman G. A. (2019). Acute Respiratory Distress Syndrome. *Nature Reviews Disease Primers*.

[3] Swenson K. E., Swenson E. R. (2021). Pathophysiology of Acute Respiratory Distress Syndrome and COVID-19 Lung Injury. *Critical Care Clinics*.

[4] Sinha P., Bos L. D. (2021). Pathophysiology of the Acute Respiratory Distress Syndrome: Insights From Clinical Studies. *Critical Care Clinics*.

[5] Sadana D., Kaur S., Sankaramangalam K. (2022). Mortality Associated With Acute Respiratory Distress Syndrome, 2009–2019: A Systematic Review and Meta-Analysis. *Critical Care and Resuscitation*.

[6] Torres L. K., Hoffman K. L., Oromendia C. (2021). Attributable Mortality of Acute Respiratory Distress Syndrome: A Systematic Review, Meta-Analysis and Survival Analysis Using Targeted Minimum Loss-Based Estimation. *Thorax*.

[7] Bellani G., Laffey J. G., Pham T. (2016). Epidemiology, Patterns of Care, and Mortality for Patients With Acute Respiratory Distress Syndrome in Intensive Care Units in 50 Countries. *JAMA*.

[8] Neto A. S., Barbas C. S. V., Simonis F. D. (2016). Epidemiological Characteristics, Practice of Ventilation, and Clinical Outcome in Patients at Risk of Acute Respiratory Distress Syndrome in Intensive Care Units From 16 Countries (Provent): An International, Multicentre, Prospective Study. *The Lancet Respiratory Medicine*.

[9] Ashbaugh D. G., Bigelow D. B., Petty T. L., Levine B. E. (1967). Acute Respiratory Distress in Adults. *Lancet*.

[10] Bernard G. R., Artigas A., Brigham K. L. (1994). The American-European Consensus Conference on ARDS. Definitions, Mechanisms, Relevant Outcomes, and Clinical Trial Coordination. *American Journal of Respiratory and Critical Care Medicine*.

[11] Ranieri V. M., Rubenfeld G. D., Thompson B. T. (2012). Acute Respiratory Distress Syndrome: The Berlin Definition. *JAMA*.

[12] Ferguson N. D., Fan E., Camporota L. (2012). The Berlin Definition of ARDS: An Expanded Rationale, Justification, and Supplementary Material. *Intensive Care Medicine*.

[13] Matthay M. A., Arabi Y., Arroliga A. C. (2024). A New Global Definition of Acute Respiratory Distress Syndrome. *American Journal of Respiratory and Critical Care Medicine*.

[14] Delong P., Murray J. A., Cook C. K. (2006). Critical Care Issues for the Nephrologist: Mechanical Ventilation in the Management of Acute Respiratory Distress Syndrome. *Seminars in Dialysis*.

[15] Parker J. C., Hernandez L. A., Peevy K. J. (1993). Mechanisms of Ventilator-Induced Lung Injury. *Critical Care Medicine*.

[16] Tremblay L. N., Slutsky A. S. (2006). Ventilator-Induced Lung Injury: From the Bench to the Bedside. *Intensive Care Medicine*.

[17] Meade M. O., Cook D. J., Guyatt G. H. (2008). Ventilation Strategy Using Low Tidal Volumes, Recruitment Maneuvers, and High Positive End-Expiratory Pressure for Acute Lung Injury and Acute Respiratory Distress Syndrome: A Randomized Controlled Trial. *JAMA*.

[18] Plataki M., Hubmayr R. D. (2010). The Physical Basis of Ventilator-Induced Lung Injury. *Expert Review of Respiratory Medicine*.

[19] Beitler J. R., Malhotra A., Thompson B. T. (2016). Ventilator-Induced Lung Injury. *Clinics in Chest Medicine*.

[20] Gattinoni L., Tonetti T., Cressoni M. (2016). Ventilator-Related Causes of Lung Injury: The Mechanical Power. *Intensive Care Medicine*.

[21] Amato M. B., Barbas C. S., Medeiros D. M. (1998). Effect of a Protective-Ventilation Strategy on Mortality in the Acute Respiratory Distress Syndrome. *New England Journal of Medicine*.

[22] Dreyfuss D., Soler P., Basset G., Saumon G. (1988). High Inflation Pressure Pulmonary Edema. Respective Effects of High Airway Pressure, High Tidal Volume, and Positive End-Expiratory Pressure. *American Review of Respiratory Disease*.

[23] Brower R. G., Matthay M. A., Morris A., Schoenfeld D., Thompson B. T., Wheeler A. (2000). Ventilation With Lower Tidal Volumes as Compared With Traditional Tidal Volumes for Acute Lung Injury and the Acute Respiratory Distress Syndrome. *New England Journal of Medicine*.

[24] Amato M. B., Meade M. O., Slutsky A. S. (2015). Driving Pressure and Survival in the Acute Respiratory Distress Syndrome. *New England Journal of Medicine*.

[25] Bendixen H. H., Whyte H., Laver M. B. (1963). Impaired Oxygenation in Surgical Patients During General Anesthesia With Controlled Ventilation. A Concept of Atelectasis. *New England Journal of Medicine*.

[26] Hickling K. G., Henderson S. J., Jackson R. (1990). Low Mortality Associated With Low Volume Pressure Limited Ventilation With Permissive Hypercapnia in Severe Adult Respiratory Distress Syndrome. *Intensive Care Medicine*.

[27] Petrucci N., Iacovelli W. (2004). Ventilation With Lower Tidal Volumes Versus Traditional Tidal Volumes in Adults for Acute Lung Injury and Acute Respiratory Distress Syndrome. *Cochrane Database of Systematic Reviews*.

[28] Briel M., Meade M., Mercat A. (2010). Higher vs. Lower Positive End-Expiratory Pressure in Patients With Acute Lung Injury and Acute Respiratory Distress Syndrome: Systematic Review and Meta-Analysis. *JAMA*.

[29] Kregenow D. A., Rubenfeld G. D., Hudson L. D., Swenson E. R. (2006). Hypercapnic Acidosis and Mortality in Acute Lung Injury. *Critical Care Medicine*.

[30] Girardis M., Busani S., Damiani E. (2016). Effect of Conservative vs. Conventional Oxygen Therapy on Mortality Among Patients in an Intensive Care Unit: The Oxygen-ICU Randomized Clinical Trial. *JAMA*.

[31] Fan E., Del Sorbo L., Goligher E. C. (2017). An Official American Thoracic Society/European Society of Intensive Care Medicine/Society of Critical Care Medicine Clinical Practice Guideline: Mechanical Ventilation in Adult Patients With Acute Respiratory Distress Syndrome. *American Journal of Respiratory and Critical Care Medicine*.

[32] Brochard L., Roudot-Thoraval F., Roupie E. (1998). Tidal Volume Reduction for Prevention of Ventilator-Induced Lung Injury in Acute Respiratory Distress Syndrome. *American Journal of Respiratory and Critical Care Medicine*.

[33] Brower R. G., Shanholtz C. B., Fessler H. E. (1999). Prospective, Randomized, Controlled Clinical Trial Comparing Traditional Versus Reduced Tidal Volume Ventilation in Acute Respiratory Distress Syndrome Patients. *Critical Care Medicine*.

[34] Stewart T. E., Meade M. O., Cook D. J. (1998). Evaluation of a Ventilation Strategy to Prevent Barotrauma in Patients at High Risk for Acute Respiratory Distress Syndrome. *New England Journal of Medicine*.

[35] Kalhan R., Mikkelsen M., Dedhiya P. (2006). Underuse of Lung Protective Ventilation: Analysis of Potential Factors to Explain Physician Behavior. *Critical Care Medicine*.

[36] Umoh N. J., Fan E., Mendez-Tellez P. A. (2008). Patient and Intensive Care Unit Organizational Factors Associated With Low Tidal Volume Ventilation in Acute Lung Injury. *Critical Care Medicine*.

[37] Young M. P., Manning H. L., Wilson D. L. (2004). Ventilation of Patients With Acute Lung Injury and Acute Respiratory Distress Syndrome: Has New Evidence Changed Clinical Practice?. *Critical Care Medicine*.

[38] Checkley W., Brower R., Korpak A., Thompson B. T. (2008). Effects of a Clinical Trial on Mechanical Ventilation Practices in Patients With Acute Lung Injury. *American Journal of Respiratory and Critical Care Medicine*.

[39] Walkey A. J., Wiener R. S. (2012). Risk Factors for Underuse of Lung-Protective Ventilation in Acute Lung Injury. *Journal of Critical Care*.

[40] Esteban A., Ferguson N. D., Meade M. O. (2008). Evolution of Mechanical Ventilation in Response to Clinical Research. *American Journal of Respiratory and Critical Care Medicine*.

[41] Spece L. J., Mitchell K. H., Caldwell E. S., Gundel S. J., Jolley S. E., Hough C. L. (2018). Low Tidal Volume Ventilation Use Remains Low in Patients With Acute Respiratory Distress Syndrome at a Single Center. *Journal of Critical Care*.

[42] Walkey A. J., Goligher E. C., Del Sorbo L. (2017). Low Tidal Volume Versus Non-Volume-Limited Strategies for Patients With Acute Respiratory Distress Syndrome. A Systematic Review and Meta-Analysis. *Annals of the American Thoracic Society*.

[43] Simonis F. D., Serpa Neto A., Binnekade J. M. (2018). Effect of a Low vs. Intermediate Tidal Volume Strategy on Ventilator-Free Days in Intensive Care Unit Patients Without ARDS: A Randomized Clinical Trial. *JAMA*.

[44] LeSieur M. N., Bosch N. A., Walkey A. J. (2023). Hospital Variation in Mortality and Ventilator Management Among Mechanically Ventilated Patients With ARDS. *Journal of Intensive Care Medicine*.

[45] Dianti J., Matelski J., Tisminetzky M. (2021). Comparing the Effects of Tidal Volume, Driving Pressure, and Mechanical Power on Mortality in Trials of Lung-Protective Mechanical Ventilation. *Respiratory Care*.

[46] De Monnin K., Terian E., Yaegar L. H. (2022). Low Tidal Volume Ventilation for Emergency Department Patients: A Systematic Review and Meta-Analysis on Practice Patterns and Clinical Impact. *Critical Care Medicine*.

[47] Tongyoo S., Viarasilpa T., Deawtrakulchai P., Subpinyo S., Suppasilp C., Permpikul C. (2024). Comparison of Limited Driving Pressure Ventilation and Low Tidal Volume Strategies in Adults With Acute Respiratory Failure on Mechanical Ventilation: A Randomized Controlled Trial. *Therapeutic Advances in Respiratory Disease*.

[48] Laffey J. G., Tanaka M., Engelberts D. (2000). Therapeutic Hypercapnia Reduces Pulmonary and Systemic Injury Following In Vivo Lung Reperfusion. *American Journal of Respiratory and Critical Care Medicine*.

[49] Yang W. C., Song C. Y., Wang N. (2013). Hypercapnic Acidosis Confers Antioxidant and Anti-Apoptosis Effects Against Ventilator-Induced Lung Injury. *Laboratory Investigation*.

[50] Hummler H. D., Banke K., Wolfson M. R. (2016). The Effects of Lung Protective Ventilation or Hypercapnic Acidosis on Gas Exchange and Lung Injury in Surfactant Deficient Rabbits. *PLoS One*.

[51] Briva A., Vadász I., Lecuona E. (2007). High CO_2_ Levels Impair Alveolar Epithelial Function Independently of pH. *PLoS One*.

[52] Vadász I., Dada L. A., Briva A. (2012). Evolutionary Conserved Role of c-Jun-N-terminal Kinase in CO_2_-Induced Epithelial Dysfunction. *PLoS One*.

[53] Dada L. A., Trejo Bittar H. E., Welch L. C. (2015). High CO_2_ Leads to Na, K-ATPase Endocytosis via c-Jun Amino-Terminal Kinase-Induced LMO_7_b Phosphorylation. *Molecular and Cellular Biology*.

[54] Nin N., Muriel A., Peñuelas O. (2017). Severe Hypercapnia and Outcome of Mechanically Ventilated Patients With Moderate or Severe Acute Respiratory Distress Syndrome. *Intensive Care Medicine*.

[55] Tiruvoipati R., Pilcher D., Buscher H., Botha J., Bailey M. (2017). Effects of Hypercapnia and Hypercapnic Acidosis on Hospital Mortality in Mechanically Ventilated Patients. *Critical Care Medicine*.

[56] Tonetti T., Vasques F., Rapetti F. (2017). Driving Pressure and Mechanical Power: New Targets for VILI Prevention. *Annals of Translational Medicine*.

[57] Guérin C., Papazian L., Reignier J., Ayzac L., Loundou A., Forel J. M. (2016). Effect of Driving Pressure on Mortality in ARDS Patients During Lung Protective Mechanical Ventilation in Two Randomized Controlled Trials. *Critical Care*.

[58] Aoyama H., Pettenuzzo T., Aoyama K., Pinto R., Englesakis M., Fan E. (2018). Association of Driving Pressure With Mortality Among Ventilated Patients With Acute Respiratory Distress Syndrome: A Systematic Review and Meta-Analysis. *Critical Care Medicine*.

[59] Sahetya S. K., Mallow C., Sevransky J. E. (2019). Association Between Hospital Mortality and Inspiratory Airway Pressures in Mechanically Ventilated Patients Without Acute Respiratory Distress Syndrome: A Prospective Cohort Study. *Critical Care*.

[60] Pereira Romano M. L., Maia I. S., Laranjeira L. N. (2020). Driving Pressure-Limited Strategy for Patients With Acute Respiratory Distress Syndrome. A Pilot Randomized Clinical Trial. *Annals of the American Thoracic Society*.

[61] Lanspa M. J., Peltan I. D., Jacobs J. R. (2019). Driving Pressure Is Not Associated With Mortality in Mechanically Ventilated Patients Without ARDS. *Critical Care*.

[62] Blondonnet R., Joubert E., Godet T. (2019). Driving Pressure and Acute Respiratory Distress Syndrome in Critically Ill Patients. *Respirology*.

[63] Roca O., Peñuelas O., Muriel A. (2021). Driving Pressure Is a Risk Factor for ARDS in Mechanically Ventilated Subjects Without ARDS. *Respiratory Care*.

[64] Hamama K. M., Fathy S. M., AbdAlrahman R. S., Alsherif S. E. D. I., Ahmed S. A. (2021). Driving Pressure-Guided Ventilation Versus Protective Lung Ventilation in ARDS Patients: A Prospective Randomized Controlled Study. *Egyptian Journal of Anaesthesia*.

[65] Goodwin A. J., Brinton D. L., Terry C. (2023). Driving Pressure, Elastance, and Outcomes in a Real-World Setting: A Bi-Center Analysis of Electronic Health Record Data. *Critical Care Explorations*.

[66] Park M., Yoon S., Nam J. S. (2023). Driving Pressure-Guided Ventilation and Postoperative Pulmonary Complications in Thoracic Surgery: A Multicentre Randomised Clinical Trial. *British Journal of Anaesthesia*.

[67] Neto A. S., Hemmes S. N., Barbas C. S. (2016). Association Between Driving Pressure and Development of Postoperative Pulmonary Complications in Patients Undergoing Mechanical Ventilation for General Anaesthesia: A Meta-Analysis of Individual Patient Data. *The Lancet Respiratory Medicine*.

[68] Ladha K., Vidal Melo M. F., McLean D. J. (2015). Intraoperative Protective Mechanical Ventilation and Risk of Postoperative Respiratory Complications: Hospital Based Registry Study. *BMJ*.

[69] Mathis M. R., Duggal N. M., Likosky D. S. (2019). Intraoperative Mechanical Ventilation and Postoperative Pulmonary Complications After Cardiac Surgery. *Anesthesiology*.

[70] Mazzinari G., Serpa Neto A., Hemmes S. N. T. (2021). The Association of Intraoperative Driving Pressure With Postoperative Pulmonary Complications in Open Versus Closed Abdominal Surgery Patients: A Posthoc Propensity Score-Weighted Cohort Analysis of the LAS VEGAS Study. *BMC Anesthesiology*.

[71] Kim Y. J., Kim B. R., Kim H. W. (2023). Effect of Driving Pressure-Guided Positive End-Expiratory Pressure on Postoperative Pulmonary Complications in Patients Undergoing Laparoscopic or Robotic Surgery: A Randomised Controlled Trial. *British Journal of Anaesthesia*.

[72] Gu W. J., Cen Y., Zhao F. Z., Wang H. J., Yin H. Y., Zheng X. F. (2024). Association Between Driving Pressure-Guided Ventilation and Postoperative Pulmonary Complications in Surgical Patients: A Meta-Analysis With Trial Sequential Analysis. *British Journal of Anaesthesia*.

[73] Muñoz J., Cedeño J. A., Castañeda G. F., Visedo L. C. (2024). Personalized Ventilation Adjustment in ARDS: A Systematic Review and Meta-Analysis of Image, Driving Pressure, Transpulmonary Pressure, and Mechanical Power. *Heart & Lung*.

[74] Urner M., Jüni P., Rojas-Saunero L. P. (2023). Limiting Dynamic Driving Pressure in Patients Requiring Mechanical Ventilation. *Critical Care Medicine*.

[75] Costa E. L. V., Slutsky A. S., Brochard L. J. (2021). Ventilatory Variables and Mechanical Power in Patients With Acute Respiratory Distress Syndrome. *American Journal of Respiratory and Critical Care Medicine*.

[76] Xie C., Tang W., Leng J., Yang P., Zhang Y., Wang S. (2024). Impacts of Initial ICU Driving Pressure on Outcomes in Acute Hypoxemic Respiratory Failure: A MIMIC-IV Database Study. *Scientific Reports*.

[77] Meyer K. C., Ershler W., Rosenthal N. S., Lu X. G., Peterson K. (1996). Immune Dysregulation in the Aging Human Lung. *American Journal of Respiratory and Critical Care Medicine*.

[78] Lai-Fook S. J., Hyatt R. E. (2000). Effects of Age on Elastic Moduli of Human Lungs. *Journal of Applied Physiology*.

[79] Kao K. C., Hsieh M. J., Lin S. W. (2018). Survival Predictors in Elderly Patients With Acute Respiratory Distress Syndrome: A Prospective Observational Cohort Study. *Scientific Reports*.

[80] Luo L., Shaver C. M., Zhao Z. (2017). Clinical Predictors of Hospital Mortality Differ Between Direct and Indirect ARDS. *Chest*.

[81] Papoutsi E., Gkirgkiris K., Tsolaki V. (2024). Association Between Baseline Driving Pressure and Mortality in Very Old Patients With Acute Respiratory Distress Syndrome. *American Journal of Respiratory and Critical Care Medicine*.

[82] De Jong A., Cossic J., Verzilli D. (2018). Impact of the Driving Pressure on Mortality in Obese and Non-Obese ARDS Patients: A Retrospective Study of 362 Cases. *Intensive Care Medicine*.

[83] Yoshida T., Fujino Y., Amato M. B., Kavanagh B. P. (2017). Fifty Years of Research in ARDS. Spontaneous Breathing During Mechanical Ventilation. Risks, Mechanisms, and Management. *American Journal of Respiratory and Critical Care Medicine*.

[84] Zhou Y., Holets S. R., Li M. (2021). Etiology, Incidence, and Outcomes of Patient-Ventilator Asynchrony in Critically-Ill Patients Undergoing Invasive Mechanical Ventilation. *Scientific Reports*.

[85] Sassoon C. S., Foster G. T. (2001). Patient-Ventilator Asynchrony. *Current Opinion in Critical Care*.

[86] Maia I. S., Medrado F. A., Tramujas L. (2024). Prospective, Randomized, Controlled Trial Assessing the Effects of a Driving Pressure-Limiting Strategy for Patients With Acute Respiratory Distress Syndrome due to Community-Acquired Pneumonia (STAMINA Trial): Protocol and Statistical Analysis Plan. *Critical Care Science*.

[87] Zhou Y., Cheng J., Zhu S. (2024). Early Pathophysiology-Driven Airway Pressure Release Ventilation Versus Low Tidal Volume Ventilation Strategy for Patients With Moderate-Severe ARDS: Study Protocol for a Randomized, Multicenter, Controlled Trial. *BMC Pulmonary Medicine*.

